# Human-specific protein-coding and lncRNA genes cast sex-biased genes in the brain and their relationships with brain diseases

**DOI:** 10.1186/s13293-024-00659-3

**Published:** 2024-10-29

**Authors:** Sha He, Xuecong Zhang, Hao Zhu

**Affiliations:** 1 Bioinformatics Section, School of Basic Medical Sciences, Southern Medical University, Guangzhou, 510515 China; 2 Guangdong-Hong Kong-Macao Greater Bay Area Center for Brain Science and Brain-Inspired Intelligence, Southern Medical University, Guangzhou, 510515 China; 3 Guangdong Provincial Key Lab of Single Cell Technology and Application, Southern Medical University, Guangzhou, 510515 China; 4 Shenzhen Clinical Research Center for Tuberculosis, National Clinical Research Center for Infectious Diseases, Shenzhen Third People’s Hospital, Shenzhen, Guangdong China

## Abstract

**Background:**

Gene expression shows sex bias in the brain as it does in other organs. Since female and male humans exhibit noticeable differences in emotions, logical thinking, movement, spatial orientation, and even the incidence of neurological disorders, sex biases in the brain are especially interesting, but how they are determined, whether they are conserved or lineage specific, and what the consequences of the biases are, remain poorly explored and understood.

**Methods:**

Based on RNA-seq datasets from 16  and 14 brain regions in humans and macaques across developmental periods and from patients with brain diseases, we used linear mixed models (LMMs) to differentiate variations in gene expression caused by factors of interest and confounding factors and identify four types of sex-biased genes. Effect size and confidence in each effect were measured upon the local false sign rate (LFSR). We utilized the *biomaRt* R package to acquire orthologous genes in humans and macaques from the BioMart Ensembl website. Transcriptional regulation of sex-biased genes by sex hormones and lncRNAs were analyzed using the *CellOracle*, *GENIE3*, and *Longtarget* programs. Sex-biased genes’ functions were revealed by gene set enrichment analysis using multiple methods.

**Results:**

Lineage-specific sex-biased genes greatly determine the distinct sex biases in human and macaque brains. In humans, those encoding proteins contribute directly to immune-related functions, and those encoding lncRNAs intensively regulate the expression of other sex-biased genes, especially genes with immune-related functions. The identified sex-specific differentially expressed genes (ssDEGs) upon gene expression in disease and normal samples also indicate that protein-coding ssDEGs are conserved in humans and macaques but that lncRNA ssDEGs are not conserved. The results answer the above questions, reveal an intrinsic relationship between sex biases in the brain and sex-biased susceptibility to brain diseases, and will help researchers investigate human- and sex-specific ncRNA targets for brain diseases.

**Conclusions:**

Human-specific genes greatly cast sex-biased genes in the brain and their relationships with brain diseases, with protein-coding genes contributing to immune response related functions and lncRNA genes critically regulating sex-biased genes. The high proportions of lineage-specific lncRNAs in mammalian genomes indicate that sex biases may have evolved rapidly in not only the brain but also other organs.

**Supplementary Information:**

The online version contains supplementary material available at 10.1186/s13293-024-00659-3.

## Abstract (plain language summary)

All animals show differences between the sexes, which are called sex biases. The human brain, as the most complex organ, critically differs between humans and other primates, and female and male humans exhibit noticeable differences in emotions, logical thinking, movement, spatial orientation, and even the incidence of neurological disorders, indicating significant sex biases in the brain. These differences are intriguing to both researchers and ordinary people and await investigation. This study addressed key questions on sex bias in the brain by exploring datasets from human and macaque brains and from patients with brain diseases. Our results revealed that the cross-sex differences in gene expression in human and macaque brains are strongly regulated by lineage-specific lncRNAs, and this regulation makes sex bias in the brain highly lineage specific. In particular, both target genes of human-specific lncRNAs and human-specific protein-coding genes are enriched in immune-related genes, indicating that immune-related functions show sex bias in humans but not in macaques. These results have significant implications, as they explain the relationships between sex-biased genes and brain diseases and the rapid evolution of sex bias in the brain (and other organs), and highlight sex-specific targets for brain diseases.

**Supplementary Information:**

The online version contains supplementary material available at 10.1186/s13293-024-00659-3.

## Highlights

**•** Many genes show sex-biased expression in human and macaque brains across regions and developmental ages. Those encoding proteins are conserved across humans and macaques, but those encoding lncRNAs are not.
**•** Most sex-biased lncRNA genes are lineage-specific and regulate sex-biased protein-coding genes lineage-specifically; notably, the lncRNA target genes are enriched for immune-related functions in humans but not in macaques.
**•** Furthermore, among the sex-biased protein-coding genes, those that are human-specific are also enriched for immune-related functions.
**•** These findings reveal key traits and explain the rapid evolution of sex bias in the brain, explain sex-biased susceptibility to brain diseases, and highlight human-specific lncRNAs as sex-specific targets for brain diseases.

**Supplementary Information:**

The online version contains supplementary material available at 10.1186/s13293-024-00659-3.

## Introduction

Increasing evidence indicates that sex differences in the brain influence not only brain functions (e.g., spatial learning, nonverbal reasoning, fear, and anxiety) [1] but also the incidence, development, and therapeutic effects of brain diseases [2]. The incidence of chronic neurodegenerative diseases is reportedly 1.42 times greater in males than in females [3], and sex-specific treatment can improve the prognosis of glioblastoma patients [4]. However, the genetic basis of sex differences in the brain remains poorly understood, especially whether the differences are exclusive to humans or conserved in primates.

Many genes exhibit sex-specific expression (called sex-biased genes) in human organs [5–8]. Studies have also examined sex-biased gene expression in organs across mammals [7, 8]. With respect to sex-biased genes in the brain, previous studies focused on the cortex, protein-coding genes, and gene expression in adulthood. However, the brain comprises many regions with distinct structures and functions, and gene expression in the brain evolves spatiotemporally [9]. These findings explain why conclusions from previous studies seem rather inconsistent. For example, many genes exhibit conserved sex-biased expression across mammals, with most sex-biased expression occurring early in mammalian evolution [7], and at the same time, gene expression shows distinct sex bias across mammals and organs, with fast evolution of sex-biased gene expression [8]. These inconsistencies occur because the mechanisms that determine conservation and the factors that drive rapid evolution remain unclear. In particular, lncRNAs, which can intensively regulate gene expression quite species-specifically, have been overlooked in previous studies. Many lncRNA and epigenetic studies have revealed the regulatory functions of lncRNAs [10, 11]. Sex-biased methylated genomic regions have been detected in postmortem brain samples from patients with psychiatric disorders [12], but the impacts of lncRNAs on sex bias in the brain and sex bias in brain diseases remain understudied [13, 14]. The expression of the lncRNA LINC00473, which is decreased in female patients with depression, provides a notable example and highlights that lncRNAs may critically link sex bias in the brain and sex-biased features of brain diseases [15]. The gaps in the understanding of sex bias in the brain and the inconsistent conclusions of previous studies call for brain-, brain disease-, and lncRNA-centered sex bias analyses.

With respect to sex bias in the brain, three questions are of special interest: To what extent does transcriptional regulation by lncRNAs influence the bias? To what extent is the bias human-specific? Does the bias have a relationship with brain diseases? This study addressed these questions by analyzing RNA-seq datasets from 16 human brain regions across four developmental periods, 14 corresponding macaque brain regions across two corresponding developmental periods, and patients with brain diseases (together with the corresponding healthy individuals). The brain diseases were schizophrenia (SCZ), autism spectrum disorder (ASD), low-grade glioma (LGG), and glioblastoma (GBM). We identified four types of sex-biased genes, and 7647 genes whose expression showed spatiotemporal variation across brain regions and developmental periods were identified as “spatiotemporal-specific sex-biased genes”. Sex-biased genes are enriched for neurogenesis- and immune response-related functions in humans but only for neurogenesis-related functions in macaques. Notably, few sex-biased genes, especially lncRNA genes, are shared between human and macaque brains, which is consistent with rapid evolution observations [8], and sex-based genes are enriched in targets of species-specific lncRNAs, which has not been previously reported and is also consistent with the rapid evolution observations. We also found that human-specific protein-coding genes contribute directly to sex-biased immune-related functions in the human brain.

## Materials and methods

### Data collection

RNA-seq data were collected from multiple resources, including 510 human samples covering four developmental periods (fetal period, childhood, adolescence, and adulthood) and 16 brain regions from the psychENCODE website (http://development.psychencode.org) [16], 176 macaque samples covering two developmental periods (5 years old, 10 years old) and 14 brain regions from the GEO website (accession GSE128537) [17], and samples and controls from patients with brain diseases from public databases (Supplementary Table 1). The 16 human brain regions included the neocortex (A1C, DFC, IPC, ITC, M1C, MFC, OFC, S1C, STC, V1C, and VFC), amygdala (AMY), cerebellar cortex (CBC), hippocampus (HIP), thalamus (MD), and striatum (STR) (Supplementary Table 2), and the 14 macaque brain regions are their equivalent. These two macaque developmental periods correspond to adolescence and adulthood in humans [18]. On average, each region has 7.9 samples at each developmental period. Gene expression data are in the form of count matrices.

The brain disease data were obtained from patients with schizophrenia (SCZ) [19], autism spectrum disorder (ASD) [20], and brain tumors (low-grade glioma, LGG, and glioblastoma, GBM) (httts://xena.ucsc.edu). The UCSC Xena database is built upon The Cancer Genome Atlas (TCGA) database and the GTEx database [21, 22] by reprocessing data and removing batch effects (https://xenabrowser.net/datapages/) [23]. We used the Gene Expression Profiling Interactive Analysis (GEPIA2) platform, which establishes the tissue matching information between the above two databases [24], to obtain 152 GBM tumor samples, 508 LGG tumor samples, and 206 normal brain samples (derived from the cortex and frontal cortex regions). The SCZ RNA-seq data were obtained from the http://eqtl.brainseq.org/phase2/ website. The data cover DFC and HIP regions [19]; the DFC data comprises 149 diseased and 210 normal samples, and the HIP data comprises 130 diseased and 228 normal samples. The ASD RNA-seq data were obtained from the https://www.synapse.org/#!Synapse:syn4587609 website and included 45 diseased and 40 normal samples from the adult brain [20].

### Identification of sex-biased genes and sex-biased gene expression

Multiple factors (e.g., sex and age) influence the regulation of tissue- and organ-specific gene expression. When examining some factors that exert “fixed effects”, other factors may become confounding factors. Thus, the effects of different kinds of factors should be properly estimated because confounding factors may generate misleading results. Among the multiple methods developed to handle different factors, linear mixed models (LMMs) can powerfully differentiate variations in gene expression caused by factors of interest and confounding factors [25–27]. We therefore used the LMM to identify four types of sex-biased genes with different factors of interest and confounding factors.

First, “spatiotemporal-specific sex-biased genes” are genes showing spatiotemporal-specific sex-biased expression. To use the LMM to detect these genes, we combined the “age” and “sex” factors in the LMM equation into an integrated categorical variable called “AgeSex” (which has 4*2 = 8 values, including fetus-female, fetus-male, childhood-female, childhood-male, adolescent-female, adolescent-male, adulthood-female, and adulthood-male). We subsequently applied this LMM to samples from one brain region using the *voom* function in the *limma* R package [28]:

$$\:\text{Y}\:\sim\:{\beta\:}_{0}+{\beta\:}_{1}\text{A}\text{g}\text{e}\text{S}\text{e}\text{x}+{\beta\:}_{2}\text{P}\text{M}\text{I}+{\beta\:}_{3}\text{R}\text{I}\text{N}+{\beta\:}_{4}\text{S}\text{i}\text{t}\text{e}+\epsilon\:.\:$$

Here, Y indicates the gene expression level, the postmortem interval (PMI) and the RNA integrity number (RIN) were treated as fixed effects, and the sequencing processing site (Site) was treated as a random effect. We used male samples from the same period and region as the reference to detect sex-biased genes for each period and region, yielding 16*4 = 64 gene sets. The union of the 64 gene sets showing sex-biased expression in at least one region or period contained 7647 genes (Supplementary Table 3). Second, “period-specific sex-biased genes” were defined as genes showing sex-biased expression in a specific period. To detect these genes, we fitted the following LMM to samples from all regions in the same period:

$$\begin {aligned}\text{Y}\:\sim\:{\beta\:}_{0} & +{\beta\:}_{1}\text{S}\text{e}\text{x}+{\beta\:}_{2}\text{R}\text{e}\text{g}\text{i}\text{o}\text{n}\\ & \quad +{\beta\:}_{3}\text{P}\text{M}\text{I}+{\beta\:}_{4}\text{R}\text{I}\text{N}+{\beta\:}_{5}\text{S}\text{i}\text{t}\text{e}+\epsilon\:.\end {aligned}$$

In this situation, since samples of a period come from all regions but only period-specific sex-biased expression was considered, “region” is a confounding factor. Thus, “Region,” “PMI,” and “RIN” were treated as fixed effects, and “Site” was treated as a random effect as before. This model yielded four sets of genes (Supplementary Table 4). Third, “region-specific sex-biased genes” were defined as genes showing region-specific sex-biased expression. To detect these genes, we fitted the following LMM to samples from one brain region:

$$\begin {aligned}\text{Y}\:\sim\:{\beta\:}_{0} & +{\beta\:}_{1}\text{S}\text{e}\text{x}+{\beta\:}_{2}\text{A}\text{g}\text{e}+{\beta\:}_{3}\text{P}\text{M}\text{I}\\ & \quad +{\beta\:}_{4}\text{R}\text{I}\text{N}+{\beta\:}_{5}\text{S}\text{i}\text{t}\text{e}+\epsilon\:.\end {aligned}$$

In this situation, since samples in a region come from all periods, “age” is a confounding factor. Thus, “Age,” “PMI,” and “RIN” were treated as fixed effects, and “Site” was treated as a random effect. This model yielded 16 sets of genes (Supplementary Table 5). Fourth, “consistently sex-biased genes” were defined as genes showing consistent sex-biased expression across regions and periods. To detect these genes, we fitted the following LMM to samples from all regions and periods:

$$\begin {aligned}\text{Y}\:\sim\:{\beta\:}_{0} & +{\beta\:}_{1}\text{S}\text{e}\text{x}+{\beta\:}_{2}\text{A}\text{g}\text{e}+{\beta\:}_{3}\text{R}\text{e}\text{g}\text{i}\text{o}\text{n}\\ & \quad +{\beta\:}_{4}\text{P}\text{M}\text{I}+{\beta\:}_{5}\text{R}\text{I}\text{N}+{\beta\:}_{6}\text{S}\text{i}\text{t}\text{e}+\epsilon\:.\end {aligned}$$

In this situation, since “consistently” means consistent across regions and periods, “region” and “age” were controlled as confounding factors. Thus, “Age,” “Region,” “PMI,” and “RIN” were treated as fixed effects, and “Site” was treated as a random effect as before. This model yielded one set of genes.

For multifactor (multivariable) systems, the false discovery rate (FDR) was proposed for correcting multiple testing [29]. The local FDR (LFDR) is an improved method that can measure the significance of specific observations (e.g., LFDR *j* denotes the probability that effect *j* would be a false discovery) [30]. Recently, the importance of estimating the size of effects has been acknowledged, and many measures of effect size, including the standard mean difference, odds ratio (OA), and Cohen’s d, have been developed. It is proposed that measuring confidence in the *sign* of each effect matters more than the confidence in each effect being nonzero, because being confident in the sign of an effect logically implies that we are confident it is nonzero [31]. With this notation, the local false sign rate (LFSR) method was developed, which takes two inputs—an effect size estimate and the corresponding standard error—rather than the usually used *p* value or *z* score [31]. The application of LFSR to genomic data analysis suggests that LFSR outperforms LFDR [32]. In theory, LFSR, which measures both effect size and confidence in each effect and combines the calculation of the two, is preferable to LFDR. Furthermore, both the fold change and the coefficients of the LMM reflect the effect size (i.e., estimation of the effect size). For these reasons, we used LFSR to compute both the effect size and significance under multiple conditions via the *mashr* R package developed by Stephens’ team [32]. “beta_matrix” and “se_matrix”, the coefficient matrix and standard error matrix derived from the fitted LMM, are two inputs to the *mashr* function *mash_set_data()* (i.e., *mash_set_data(beta_matrix, se_matrix)*).

For the sets of spatiotemporal-specific sex-biased genes, we performed a cross-region meta-analysis for each period via the *mashr* package, which allowed us to correct multiple testing across regions and periods. Genes with |log2FC|>1.0 and LFSR < 0.001 were defined as “spatiotemporal-specific sex-biased genes”. Owing to the small sample size from each brain region, we also performed a cross-region meta-analysis to identify region-specific sex-biased genes. Genes with |log2FC|>1.0 and LFSR < 0.001 were defined as region-specific sex-biased genes. Note that LFSR < 0.001 is much smaller than the normal LFSR threshold of 0.05 [31], which effectively reduces the occurrence of false positives. Moreover, |log2FC|>1.0 also reflects a large effect size.

We used |log2FC|>1.0 and FDR < 0.03 to identify “period-specific sex-biased genes” because age seems to be a more significant covariate of sex-biased gene expression than region and could make *mashr* generate a high false discovery rate. FDR = 0.03 (smaller than the popular threshold of 0.05) was used because the datasets from each period were much larger than those from each region. To identify “consistently sex-biased genes”, we (a) estimated a reasonable FDR threshold by searching the parameter space of “1.0 ≤|log2FC| ≤ 2.0 and 0.001 ≤ FDR ≤ 0.05” and (b) used Fisher’s exact test to ensure that the identified genes overlapped significantly with the union of region-, period-, and spatiotemporal-specific sex-biased genes. Genes with |log2FC|>1.0 with FDR < 0.045 were defined as consistently sex-biased genes.

Finally, the same LMM models and LFSR method were used to identify sex-biased genes in the macaque brain. Spatiotemporal-specific sex-biased genes were identified with thresholds of |log2FC|>1.0 and LFSR < 0.005 (Supplementary Table 11).

### Identification of sex-related coexpression modules

Weighted gene coexpression network analysis (*WGCNA*) is a method and program widely used to analyze gene expression patterns across samples (especially small samples) [33]. Assuming that sex-related genes have correlated expression, we applied *consensus network analysis* via *WGCNA* to 16 sets and 4 sets of region- and period-specific sex-biased genes to detect sex-related coexpression modules shared across regions or periods (called consensus modules). Consensus coexpression network analysis (i.e., consensus module analysis) revealed the structural properties of the networks and modules. The following steps were used to detect period-specific sex-related modules (default parameters were used unless otherwise stated). First, we used the *pickSoftThreshold* function to determine the soft-thresholding power, and 9 was identified as the best threshold. Second, we used the *TOMsimilarity* function to calculate the topological overlap matrix (TOM) for each period and used the *pmean* function to calculate the consensus TOM across the four periods. Third, we used the *cutreeDynamic* function (*minClusterSize* = 80 and *cutHeight* = 0.995) to identify coexpression modules. Fourth, we used the *multiSetME* function to extract module eigengenes (MEs) from each module. Finally, a module was identified as a period-specific sex-related module if it contained > 5% sex-biased genes and met at least one of the two following criteria: (a) Fisher’s exact test indicated that for genes specific to the period, sex-biased genes were significantly more enriched in the module than non-sex-biased genes (*P* < 0.05), (b) linear regression analysis of module eigengenes via the following LMM model indicated that the coefficient of the “Sex” term was significant (*P* < 0.05 and R-squared > 0.4):

$$\:\text{Y}\sim{\beta\:}_{0}+{\beta\:}_{1}\text{R}\text{e}\text{g}\text{i}\text{o}\text{n}+{\beta\:}_{2}\text{S}\text{e}\text{x}+{\beta\:}_{3}\text{P}\text{M}\text{I}+{\beta\:}_{4}\text{R}\text{I}\text{N}+\epsilon\:.$$

Here, R-squared is a statistical measure of fit that indicates how much variation in the dependent variable is explained by the independent variables. An R-squared of > 0.3 is assumed to be sufficient if there is extreme variability in the dataset; here, gene expression varies greatly across regions and sexes.

The same steps, parameters, and thresholds were used to detect region-specific sex-related modules.

### Analysis of transcriptional regulation by sex hormones

Sex hormone receptors include estrogen receptor 1 (ESR1), estrogen receptor 2 (ESR2), and androgen receptor (AR). These receptors are also ligand-activated transcription factors. First, we examined the expression of AR, ESR1, and ESR2 in the human brain by using the *gam* function in the *mgcv* R package to fit their expression levels to developmental periods. Second, we validated their expression levels in brain regions using data from the GTEx project and publicly available scRNA-seq data [16]. Third, we identified these sex hormone receptors’ transcriptional target genes by using the *CellOracle* program to scan these sex hormone receptors’ DNA-binding sites (DBSs) in the promoter regions of genes in the module (threshold = 17, 1.5 kb upstream and downstream of the transcription start site, TSS) [34]. The DNA binding motifs of sex hormone receptors were extracted from the *CIS-BP* database (http://cisbp.ccbr.utoronto.ca/index.php) [35].

To examine whether lncRNA genes with sex-biased expression are regulated by sex hormone receptors, we used Fisher’s exact test (*P* < 0.05) to assess whether the promoter regions of lncRNA genes are enriched with DBSs of sex hormone receptors.

### Analysis of transcriptional regulation by lncRNAs

On the basis of the *WGCNA*-identified coexpression modules, we jointly used the *GENIE3* and *LongTarget* programs to analyze the transcriptional regulation of sex-biased genes by the lncRNAs in each module. Since some coexpression modules were very large, we first used the *GENIE3* program to screen putative regulators (lncRNAs) and targets (sex-biased genes). *GENIE3* predicts a regulatory network between regulators and targets using a tree ensemble-based gene network inference algorithm [36]. For genes in each module, *GENIE3* identified a list of lncRNAs for each gene, with ranks indicating the probability that the former would regulate the latter. The likely regulatory relationships were identified on the basis of the following criteria: (a) the targets belonging to the top 30% for each lncRNA and (b) the lncRNAs belonging to the top 30% for each target, which has been popularly adopted by regulatory network analysis to ensure reliability [37].

Since lncRNAs can epigenetically regulate gene expression by binding to gene regulatory sequences (especially promoters) and recruiting DNA and histone modification enzymes to these binding sites to establish epigenetic modification markers, we predicted the DBS of lncRNAs in promoter regions (5 kb upstream and downstream of the TSS) of sex-biased genes using the *LongTarget* program [38, 39]. The likely regulatory relationship was defined as *binding affinity* greater than 60 (corresponding to DBS length > = 90 bp) (Supplementary Table 9).

Finally, we obtained the most likely regulatory relationships between the lncRNAs and sex-biased genes by integrating the results of *GENIE3* and *LongTarget*. We also investigated whether sex-biased lncRNA genes are more likely to colocalize with sex-biased protein-coding genes than with non-sex-biased lncRNA genes and found that for most sex-biased genes, the likelihood is high (Fisher’s exact test, FDR < < 0.05) (Supplementary Table 10).

### Gene set enrichment analysis

We performed overrepresentation analysis (ORA) using the *enrichGO* function in the *ClusterProfiler* package and the *gProfiler* program (the online version) and performed gene set enrichment analysis (GSEA) using the *gseGO* function in the *ClusterProfiler* package. The two kinds of analyses are called “gene set enrichment analysis” in the Results section but are called ORA and GSEA here to clearly describe their use.

First, to reveal the biological functions of spatiotemporal-specific sex-biased genes, we sorted genes according to log2FC values (female-biased genes have log2FC > 0, and male-biased genes have log2FC < 0). We then performed GSEA using the *gseGO* function and the Gene Ontology (GO) database [40]. GO terms with an FDR < 0.05 were considered significantly enriched. Enriched GO terms with a negative normalized enrichment score (NES) indicated enrichment of male-biased genes, and enriched GO terms with a positive NES indicated enrichment of female-biased genes (Supplementary Table 6).

**Fig. 1:**
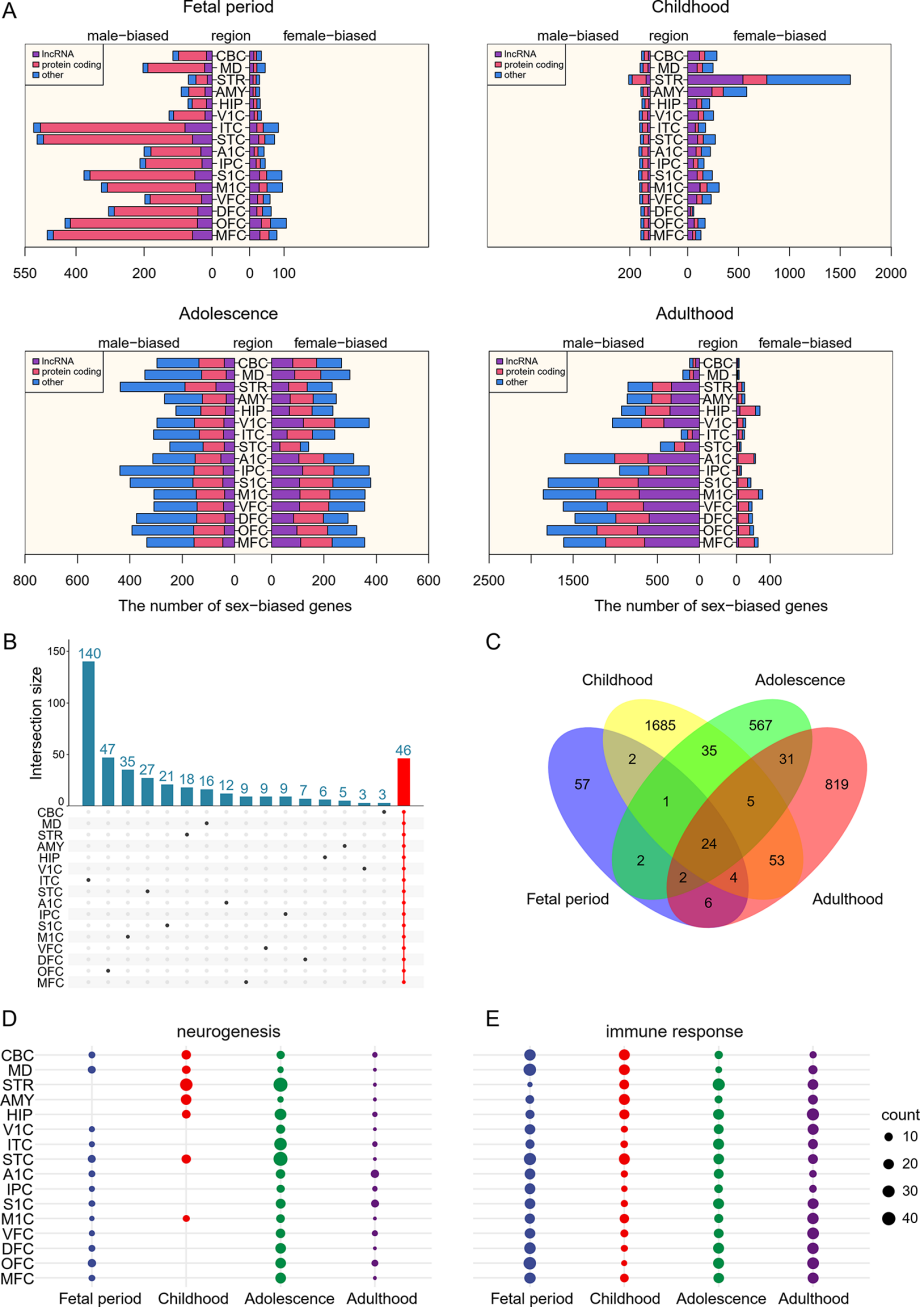
Spatiotemporally specific expression of sex-biased genes in the human brain. (**A**) Numbers of sex-biased genes in the 16 regions and four periods. More genes show male-biased expression in all regions in the fetal and adult periods, and more lncRNA genes show male-biased expression in adulthood than in other periods. (**B**) Numbers of region-specific sex-biased genes in the fetal period. (**C**) Numbers of period-specific sex-biased genes in the STR region. (**D**) The enrichment of spatiotemporal-specific sex-biased genes in the subterms of the “neurogenesis” GO term. (**E**) The enrichment of spatiotemporal-specific sex-biased genes in terms of the “immune response” GO term. In (DE), the dot size indicates the number of subterms

Second, to reveal the functions of the consensus coexpression module of period-specific sex-biased genes and region-specific sex-biased genes (gene lists without weights), we performed ORA using the *enrichGO* function and the GO database (Supplementary Tables 7, 8). GO terms with p.adjust < 0.05 were considered significantly enriched. When identifying enriched GO terms, we also used the *GO.db* function in the R package to retrieve enriched GO term subtrees (subterms) in the GO database (Fig. 1D, E; Fig. 4B).

The *gProfiler* program reports “driver terms in GO” [41]. We used it to identify the enriched GO terms of human-specific (in humans but not in macaques) sex-biased protein-coding genes (Fig. 4D), enriched GO terms of consensus coexpression modules of sex-biased genes in macaques (Supplementary Table 12), and enriched GO terms of sex-specific differentially expressed genes in the four brain diseases (Supplementary Table 14).

### Identification of orthologous and species-specific genes in humans and macaques

We utilized the *biomaRt* R package to acquire orthologous genes in humans and macaques from the BioMart Ensembl website (https://mart.ensembl.org/index.html) [42]. A total of 22,887 one-to-one orthologous protein-coding genes were identified, but no orthologous lncRNA genes were found. Therefore, we manually examined homologous lncRNA genes. First, we extracted the coordinates of 14,709 human lncRNA genes from the GENCODE v21 annotation [43]. Second, we converted these coordinates from the human genome hg38 to the macaque genome rheMac10 using the *LiftOver* function on the UCSC Genome website (http://genome.ucsc.edu/cgi-bin/hgLiftOver). Third, we used the *bedtools intersect* function in the *bedtools* package to detect whether the transformed coordinates of human lncRNA genes overlapped with annotated lncRNAs in the macaque genome (https://hgdownload.soe.ucsc.edu/downloads.html). Finally, 1821 macaque lncRNA genes were identified as homologous to human lncRNA genes on the basis of the criterion of > 20% sequence overlap. We also examined whether human or macaque lncRNA genes are conserved in mammals or simian-specific (conserved in simians, including monkeys and apes) using the *LongMan* database [39].

A recent study identified orthologous genes in hundreds of placental mammals and birds (taking humans and mice as two references) [44]. We downloaded the orthologous genes between humans and macaques from the authors’ website (https://genome.senckenberg.de//download/TOGA/) and extracted the “many2zero” and “one2zero” genes, which exist exclusively in humans but not in macaques. There are no “many2zero” genes, and 185 “one2zero” genes.

### Examination of sex differences in brain diseases

The TCGA and GTEx databases contain many RNA-seq datasets of brain tumors and normal brain tissues, respectively. The UCSC Xena website reprocessed GTEx and TCGA data by removing the batch effect (http://xena.ucsc.edu) [23]. The GEPIA2 website enables gene expression analysis of the TCGA and GTEx data at the transcript level and allows researchers to compare their data with those of the TCGA and GTEx samples (http://gepia2.cancer-pku.cn/) [24]. We used these resources to examine sex differences in brain disorders and brain tumors.

To examine the sex bias among patients with brain disorders and brain tumors, we utilized the limma-voom method (the *voom* function in the *limma* package) to identify differentially expressed genes (DEGs) in diseases (comparing gene expression in female patients and female controls and in male patients and male controls) in both females and males [28]. Genes with |log_2_FC|>2.0 and FDR < 0.05 were considered DEGs for GBM and LGG [45], and genes with FDR < 0.05 (|log_2_FC| for most genes are small) were considered DEGs for SCZ and ASD [46, 47].

We also obtained male- and female-specific DEGs by comparing gene expression differences between males and females (instead of between patients and controls) (Supplementary Table 13). Using the *gProfiler* program and the GO database (Benjamini‒Hochberg FDR < 0.05) [41], we applied gene set enrichment analysis to male- and female-specific DEGs.

### Significance test, multiple testing correction, and effect size calculation

We used Fisher’s exact test to examine whether the difference between the two sexes was significant. We used the Benjamini‒Hochberg false discovery rate (FDR) with a threshold of 0.05 for multiple testing correction, especially in gene set enrichment analysis.

We used the *effectsize* R package to compute the effect size together with Fisher’s exact test. Because most Fisher exact tests examine whether the difference between sex-biased genes and non-sex-biased genes is significant, we used OR to measure the effect size. Most of the *effectsize* results agree with the Fisher exact test results, with large OA values in many cases (Figs. 3A and 5E; Supplementary Table 10; Supplementary Table 15).

**Fig. 2:**
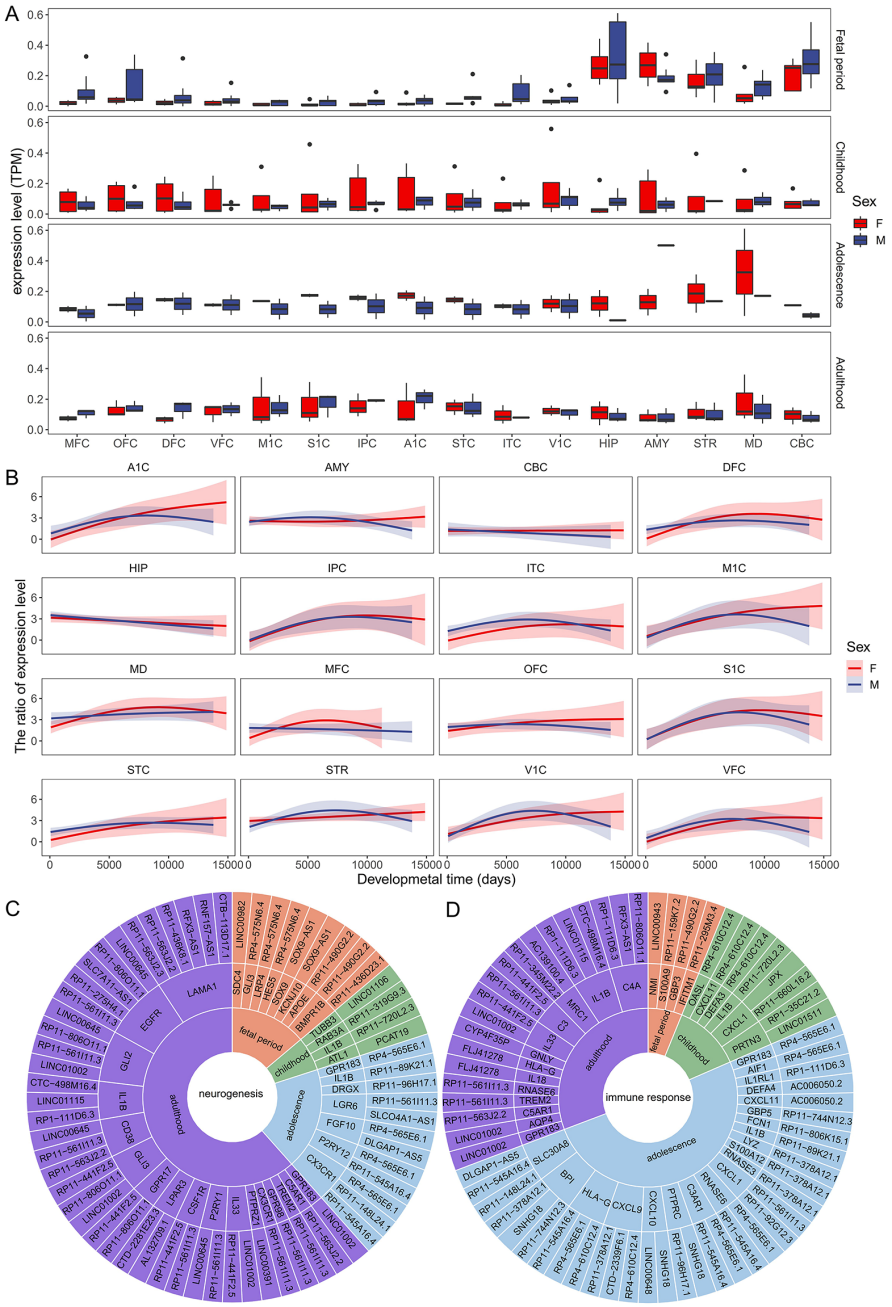
The regulation of sex-biased genes by sex hormones and lncRNAs. (**A**) AR gene expression in the 16 regions and four periods in males and females. (**B**) AR/ER expression ratios in the 16 regions during brain development in males and females. (**C**) Regulation of sex-biased genes enriched in “neurogenesis” (see Fig. 1D) by lncRNAs in the four periods. (**D**) Regulation of sex-biased genes enriched in the “immune response” (see Fig. 1E) by lncRNAs in the four periods

**Fig. 3:**
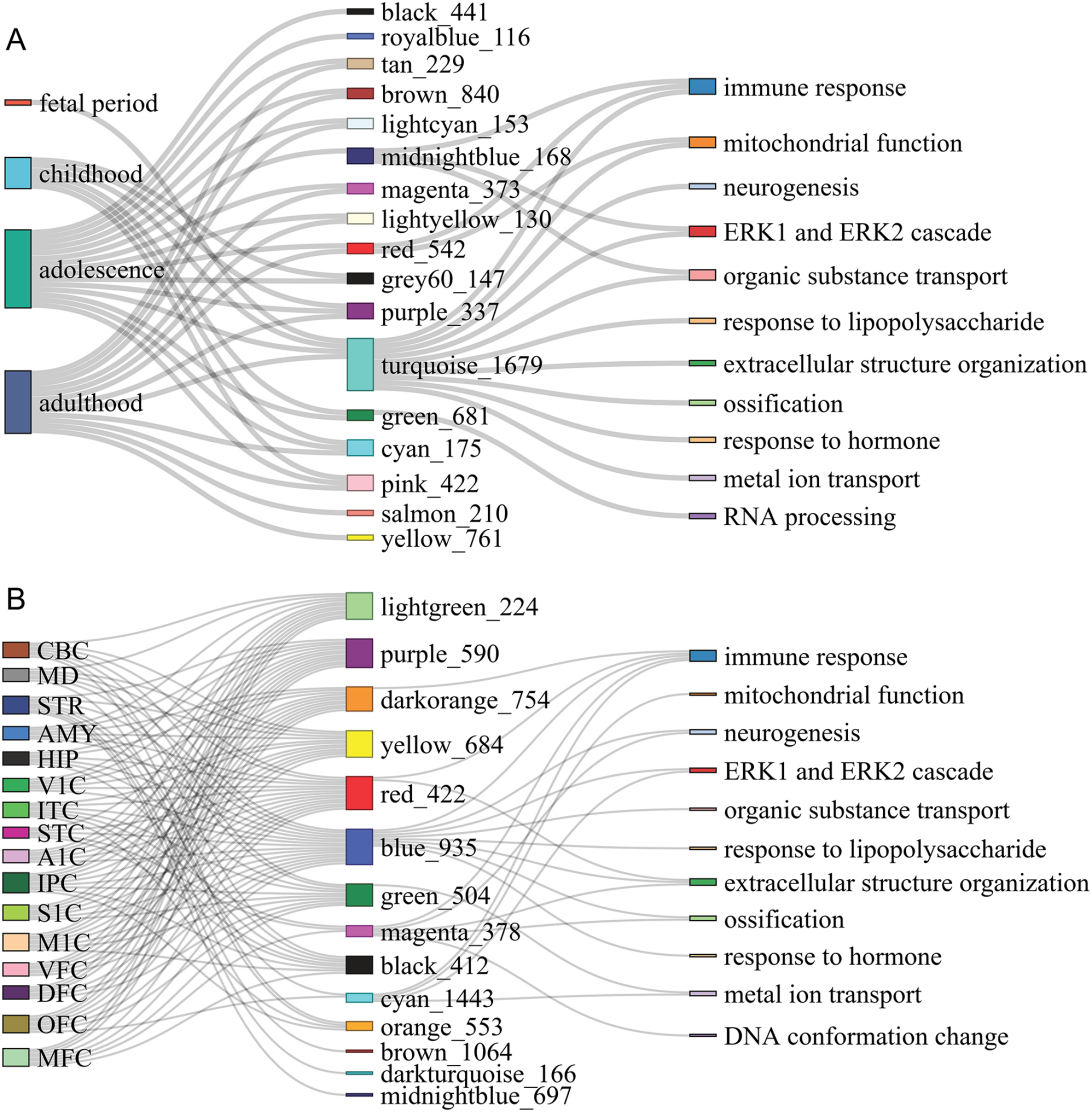
Consensus coexpression modules of period- and region-specific genes. The colors with numbers indicate modules and gene numbers. (**A**) Modules of period-specific genes and enriched GO terms. Each module shows period-specific enrichment for sex-biased genes. For example, turquoise_1679 is enriched for sex-biased genes only in fetuses (FDR = 3.0E-56, OR = 6.32), and purple_337, cyan_175, pink_442, tan_229, brown_840, and lightcyan_153 are enriched for sex-biased genes in adulthood (FDR = 4.02E14, 0.0005, 2.71E44, 2.99E69, 2.93E168, 2.54E50, OR = 2.39, 1.78, 4.17, 15.11, 8.36, 18.01). This feature is reflected by links between periods and modules. (**B**) Modules of region-specific genes and the enriched GO terms

## Results

### Sex-biased genes in the brain are enriched for neurogenesis and immune response-related functions with distinct expression patterns

To reveal sex bias in the human brain, we collected and analyzed RNA-seq data from multiple sources. The data include 510 human brain samples covering four developmental periods and 16 brain regions from the psychENCODE website (Fig. 1A) [16] and 176 macaque brain samples covering two developmental periods (5 years old and 10 years old, corresponding to adolescence and adulthood in humans) and 14 orthologous brain regions (Supplementary Table 1) [17]. These brain regions have distinct functions (Supplementary Table 2).

**Fig. 4:**
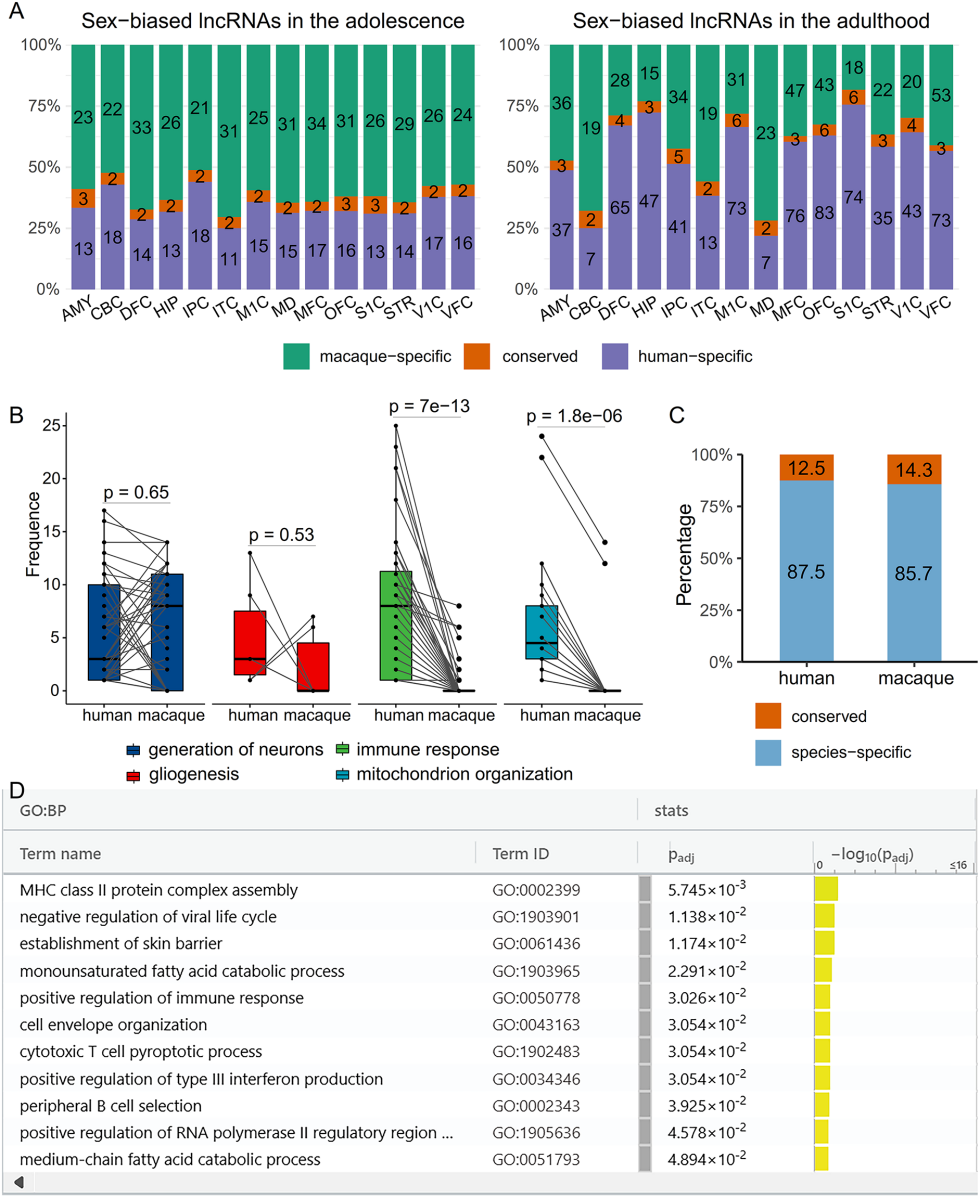
Conserved and species-specific sex-biased genes in human and macaque brains. (**A**) The numbers of conserved and species-specific sex-biased lncRNA genes in 14 brain regions in adolescence and adulthood. (**B**) The four pairs of color bars indicate the enrichment of the four GO terms in humans and macaques. Each dot in these bars indicates a sex-biased gene set that is enriched for subterms of the GO term (with the position of the dot on the Y axis indicating the number of enriched subterms). The lines between the dots in the two bars link the corresponding sex-biased gene sets. (**C**) The percentage of conserved and species-specific lncRNA genes involved in “neurogenesis”. (**D**) Genes in humans but not in macaques are enriched for immune-related GO terms. Shown are “driver terms in GO”

Since multiple factors cause gene expression variations, we used linear mixed models (LMMs) to identify sex-biased genes by controlling for the impacts of confounding factors. LMM was developed for differentiating variations in gene expression under different conditions [25–27]; thus, it allowed us to detect genes showing sex-biased expression under different conditions. A total of 7647 genes were identified as “spatiotemporal-specific sex-biased genes” (|log2FC|>1.0 and local false sign rate (LFSR) < 0.001) that presented sex-biased expression across regions and periods (Fig. 1A; Supplementary Table 3). Among these genes, 7302 are on autosomes, indicating that many autosomal genes are expressed with sex bias in the brain [8], and most show male-biased expression in the fetus and adulthood, suggesting that male-biased genes may determine the default sex bias. By treating “period” as a fixed effect and “region” as a confounding factor and treating “region” as a fixed effect and “period” as a confounding factor, we also used LMM models to identify “period-specific sex-biased genes” (|log2FC|>1.0 and FDR < 0.03) that show period-specific sex-biased expression and “region-specific sex-biased genes” (|log2FC|>1.0 and LFSR < 0.001) that show region-specific sex-biased expression (Supplementary Tables 4, 5). The number of period- and region-specific sex-biased genes that are shared across regions and regions is limited (Fig. 1BC; Supplementary Fig. 1), which is consistent with previous findings that few sex-biased genes are shared across organs [8]. By treating “region” and “period” as fixed effects, we also identified “consistently sex-biased genes” (|log_2_FC|>1.0 with FDR < 0.045) that presented consistent sex-biased expression across regions and periods. The key sex-biased gene *XIST*, whose lncRNA critically regulates X chromosome inactivation in females, was identified as a consistently sex-biased gene.

We next examined spatiotemporal-specific sex-biased genes’ biological functions using gene set enrichment analysis (the *gseGO* function in the *ClusterProfiler* package and the Gene Ontology (GO) database). Sex-biased genes in many regions in childhood and adolescence were enriched for subterms related to the “neurogenesis” GO term; however, sex-biased genes in most regions in all periods were enriched for subterms related to the “immune response” GO term (FDR < 0.05) (Fig. 1D, E). Neurogenesis and the immune response are two major aspects of brain development [48], but few studies have reported sex bias in these functions. In approximately 50% of these subterms, the enrichment of sex-biased genes shows turnover across either period or sex, suggesting complex relationships between sex-biased gene expression and brain development.

### Regulation of sex-biased genes by sex hormones and lncRNAs

Sex hormones critically regulate sexual differentiation [49], but whether they critically regulate sex-biased gene expression in the brain is less known. We first investigated whether androgen receptors (ARs) and estrogen receptors (ERs), including estradiol receptor 1 (ESR1) and estradiol receptor 2 (ESR2), directly regulate sex-biased genes. AR and ER are ligand-activated transcription factors and key players in androgen signaling and estrogen signaling [50]. We used the *CellOracle* program (threshold = 17) to scan the promoter regions (1.5 kb upstream and downstream of transcription start sites (TSSs)) of sex-biased genes and non-sex-biased genes for binding sites of AR and ER [34]. The binding sites are significantly more enriched in the promoter regions of sex-biased genes than in non-sex-biased genes (Fisher’s exact test, *P* = 0.00036, OR = 1.08), supporting the regulation of sex-biased genes by sex hormones.

To verify this conclusion, we examined AR and ER expression in the brain and found that their expression varies spatiotemporally (Fig. 2A; Supplementary Fig. 2). In many regions, the cross-sex difference is insignificant compared with the cross-region and cross-period differences (Wilcoxon rank-sum test, FDR < 0.05). We also examined the AR/ER expression ratio because the overall response of cells to sex hormones depends on this ratio [51]. In accordance with the GTEx data (Supplementary Fig. 3), the ratio, while varying spatiotemporally, is > 1.0 in both sexes in most regions. In multiple regions during early development, the ratio is greater in males than in females (Fig. 2B), suggesting that the male fetus experiences a surge of androgen to masculinize the brain [52]. In most regions during late development, the ratio is greater in females than in males, probably reflecting the changes in hormones associated with female menopause [53]. Additionally, the enrichment of multiple sex hormone-related GO terms revealed period-specific differences between males and females (Supplementary Fig. 4). The AR/ER ratio further supports the regulation of sex bias genes by sex hormones but does not satisfactorily reveal the discrepancy between sex-biased gene expression and sex hormone expression.

The above analysis revealed that the promoter regions of almost 50% of sex-biased genes lack ER and AR binding sites, indicating the regulation of these genes by other regulators. Since lncRNA genes are targets of AR and ER [54] and lncRNAs are critical transcriptional regulators in the brain [55], we next explored the regulation of sex-biased genes by lncRNAs. First, we found that the promoter regions of sex-biased lncRNA genes are significantly enriched for AR/ER binding sites compared with those of non-sex-biased genes (using *CellOralce*, Fisher’s exact test, *P* < 0.05, OR = 1.09). Second, although lncRNAs can regulate transcription *in cis* and *in trans*, we found that sex-biased lncRNA genes are significantly more likely to be located near sex-biased protein-coding genes than lncRNA genes not identified as sex biased (Fisher’s exact test, FDR < 0.05) (Supplementary Table 10). Second, we used the *LongTarget* program to examine whether the promoter regions (5 kb upstream and downstream of the TSS) of sex-biased genes contain DNA-binding sites (DBSs) of sex-biased lncRNAs [39]. We found that sex-biased genes in different regions and periods contained DBSs of distinct lncRNAs (Fig. 2C, D). These findings indicate that sex-biased genes are regulated by sex-biased lncRNAs. For example, the sex-biased gene *IL1B*, which is capable of reducing neurogenesis [56, 57], contains the DBSs of multiple lncRNAs (RP11-720L2.3, RP11-89K21.1, RP1-111D6.3, LINC01115, and CTC-498M16.4). A set of lncRNA targets with sex-biased expression is the *CXCL* family of genes, which are major mediators of inflammatory responses. RP11-561I11.3, a human-specific sex-biased lncRNA, has DBSs in *TREM2*, *CD38*, *CX3CR1*, *LGR6*, and *CSF1R* (Fig. 2C, D).

**Fig. 5:**
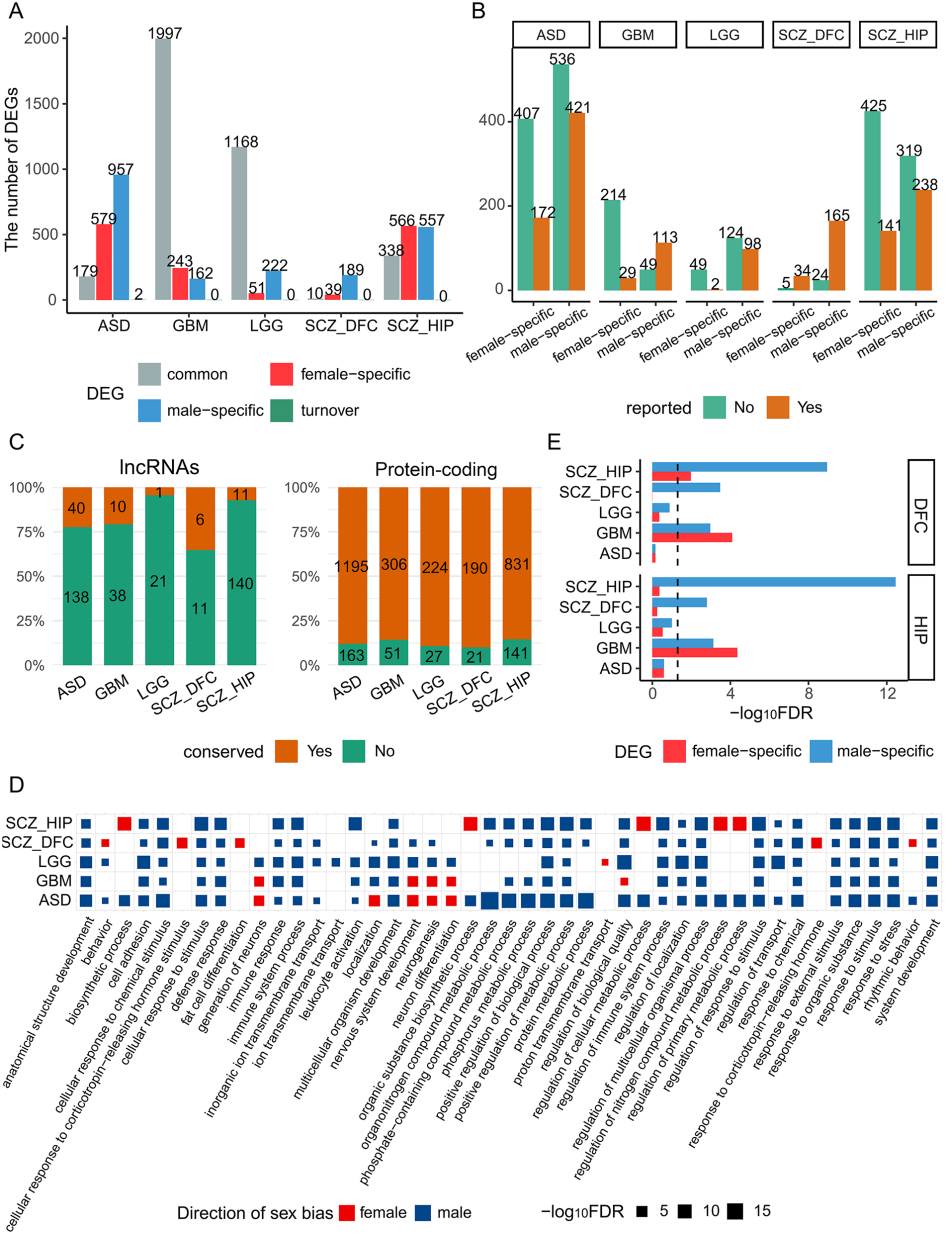
Association between sex-biased gene expression in the brain and brain diseases. (**A**) ASD and SCZ patients have higher ratios of sex-specific DEGs than GBM and LGG patients do. The bars show the numbers of common (dysregulated in the same direction in both sexes), male-specific (dysregulated only in males), female-specific (dysregulated only in females), and turnover (dysregulated in opposite directions in both sexes) sex-specific DEGs in multiple diseases. (**B**) Sex-specific DEGs that have related reports and were identified in this study. (**C**) Proportions of disease-related sex-specific DEGs conserved in humans and macaques and species specific. (**D**) The top GO terms enriched with sex-specific DEGs in the four brain diseases. The colors and square sizes indicate male/female specificity and the significance of enrichment. (**E**) Sex-specific DEGs in the four brain diseases (sampled from DFC and HIP) are enriched with sex-biased genes in DFC and HIP. The dashed line indicates the significance cutoff (FDR < 0.05), and the bars indicate male and female specificity

### Sex-biased genes and their regulators form coexpression modules

Genes and their regulators should be coexpressed to perform specific functions. To explore this feature, we used the *WGCNA* program to compare samples from the same period in males and females and identify consensus coexpression modules comprising sex-biased genes and their potential regulators. Then, with “region” treated as a confounding factor, we used an LMM model to examine whether a consensus coexpression module was period-specific sex-related (called consensus modules of period-specific genes) if the module (a) contained > 5% sex-biased genes and (b) met at least one of the two conditions: (b1) Fisher’s exact test indicates that for genes specific to the period, sex-biased genes are significantly more enriched in the module than non-sex-biased genes (*P* < 0.05), (b2) linear regression analysis of module eigengenes indicates that the coefficient of the “Sex” term was significant (*P* < 0.05 and R-squared > 0.4; see Methods). Seventeen modules were identified from the four periods (Fig. 3A; Supplementary Table 7). By applying gene set enrichment analysis (using *enrichGO* in *ClusterProfiler*) to the largest module, *turquoise_1679* (which contains 1679 genes), we revealed that this module is enriched for multiple GO terms, including “immune response”, “mitochondrial function”, and “neurogenesis” (FDR < 0.05). This result is consistent with the above-described results (Fig. 1D, E). Multiple genes in these modules, including *PTPRZ1* in adulthood and *RP1-35C21.2* in childhood, are associated with susceptibility to schizophrenia and depression [58].

We next used the same method to identify consensus modules of region-specific genes. Among the 14 modules identified from the 16 regions, 13 contained sex bias genes (Fig. 3B). The large module *blue_935* contains sex-biased genes in all regions. Notably, *blue_935* was also enriched for immune response-, mitochondrial function-, and neurogenesis-related GO terms (FDR < 0.05) (Supplementary Table 8) and shared 740 genes with *turquoise_1679*. These results suggest that immune response-related functions differ across sexes in the brain.

Given the significant presence of lncRNA genes in each coexpression module, we investigated whether lncRNAs regulate sex-biased genes within these modules. We focused on the *turquoise_1679* module, which contains 25, 44, 43, and 72 period-specific sex-biased lncRNA genes and 169, 121, 243, and 302 period-specific sex-biased other (mainly protein-coding) genes. To improve reliability, we jointly used the *GENIE3* and *LongTarget* programs to examine their regulation [36, 39]. *GENIE3* predicts the coexpression of lncRNAs and their putative targets using a tree ensemble-based gene network inference algorithm, and *LongTarget* predicts the DBSs of lncRNAs in putative target genes. In the *turquoise_1679* module, 18, 29, 34, and 59 lncRNAs have a DBS in 73, 87, 182, and 251 genes in the four periods, respectively (Supplementary Table 9), and these high ratios of the targeting relationship support that lncRNAs regulate sex bias by regulating sex-biased genes.

### Cross-species differences in sex bias are strongly affected by species-specific lncRNAs

The advanced functions of the human brain require the investigation of the extent to which sex bias in the brain is human specific. A recent study examined 14 regions in the macaque brain across the young and middle periods [17], which correspond to adolescence and adulthood in humans [18], and we therefore examined their extent on the basis of comparable gene expression data in human and macaque brains. First, using the methods described above, we identified spatiotemporal-specific sex-biased genes (|log2FC|>1.0 and LFSR < 0.005) (Supplementary Table 11) (also period- and region-specific sex-biased genes) in the macaque brain. Sex-biased gene expression shows spatiotemporal variations in the macaque brain (Supplementary Fig. 5), but the number of sex-biased genes shared between humans and macaques is limited in specific periods and regions (Fig. 4A; Supplementary Fig. 6). To exclude the possibility that the limited numbers of shared genes were caused by improper mapping between human and macaque samples, we detected consistently sex-biased genes in the macaque brain by treating “period” and “region” as confounding factors. Among the 115 consistently sex-biased genes, only 12 were also consistently sex-biased genes in humans, indicating that sex-biased genes in human and macaque brains are poorly conserved. This finding is consistent with the finding that sex-biased genes in organs (e.g., liver and kidney) in closely related mammals are poorly conserved [8].

To examine whether sex-biased genes in human and macaque brains have similar or different functions, we performed gene set enrichment analysis (*gseGO* in *ClusterProfiler*, the human GO database, FDR < 0.05) for the sex-biased gene sets from the 14 regions and two periods. To ensure cross-species comparability, we used the intersection of gene sets in the human and macaque brains and the one-to-one orthologous genes between humans and macaques. Both human and macaque genes in the intersection are enriched for “generation of neurons” and “gliogenesis”, as evidenced by the comparable numbers of gene sets in the two species enriched for subterms of the two GO terms, but only human genes in the intersection are enriched for “immune response” and “mitochondrion organization”, as evidenced by the few gene sets in macaques enriched for subterms of the two GO terms (Wilcoxon signed-rank test, *P* < 0.65, 0.53, 7E-13, 1.8E-06) (Fig. 4B). These findings indicate that immune- and mitochondria-related functions in the brain may be human specific.

These findings prompted us to identify the causes of the differences in immune- and mitochondria-related functions between human and macaque brains. We found that 80% and 87% of sex-biased lncRNA genes involved in the “immune response” and “mitochondrion function”, respectively, in the human brain are not sex-biased in the macaque brain. We also examined whether lncRNA genes involved in “neurogenesis” differ across species or are more conserved by checking the period-specific sex-biased modules in the two species. Among the four modules identified from the two macaque developmental periods, the largest brown module is enriched for neural development-related genes (Supplementary Table 12). However, when further examining the transcriptional regulation by macaque lncRNAs in these modules using the *GENIE3* and *LongTarget* programs, we found that most lncRNAs with predicted DBSs in sex-biased genes in this brown module do not have orthologs in humans. Overall, 87.5% and 85.7% of the neurogenesis-related lncRNAs are species-specific (Fig. 4C). In support of these findings, a recent study reported that microglia, astrocytes, and oligodendrocytes presented more divergent expression across species than neurons or oligodendrocyte precursor cells did [59]. These results indicate that species-specific lncRNAs intensively regulate gene expression in human and macaque brains, causing differences in sex-biased gene expression.

### Human-specific protein-coding genes in the brain have immune-related functions

The above analyses revealed 22,887 one-to-one orthologous protein-coding genes (based on the annotation in the BioMart Ensembl), 1821 homologous lncRNA genes (based on overlapping coordinates in the UCSC Genome Browser), and species-specific lncRNA genes (simply nonhomologous). To examine the contribution of human-specific protein-coding genes to sex bias in the brain, we explored the data reported by Kirilenko et al., who identified orthologous genes in hundreds of placental mammals [44]. We downloaded the genes aligned between humans and macaques (the human genome was used as a reference) from the authors’ website and extracted the “many2zero” and “one2zero” genes (i.e., genes present in humans but not in macaques). The numbers of many2zero and one2zero genes are 0 and 185, respectively. The 185 one2zero genes share many overlaps with sex-biased genes in adulthood (but not in other periods; see the “hg38-rheMac10 one2zero” column in Supplementary Table 3). Gene set enrichment analysis via the *gProfiler* program for the intersection between the one2zero genes and sex-biased genes in adulthood revealed that these genes are enriched for immune-related GO terms (Benjamini‒Hochberg FDR < 0.05) (Fig. 4D; Supplementary Fig. 7). Thus, human-specific genes, both protein-coding genes and lncRNA genes, critically determine sex bias in immune-related functions in the human brain.

### Many sex-biased genes in the brain confer susceptibility to brain diseases

Studies have investigated not only sex bias in the brain but also sex-biased characteristics of brain diseases [2]. In particular, studies have reported that immune-related genes influence sex-biased susceptibility to brain diseases [60], raising the question of whether sex bias in the brain and brain diseases have inherent relationships.

To address this question, we first identified sex-specific differentially expressed genes (DEGs) upon gene expression in disease and normal samples from the same sex in four brain diseases, including glioblastoma multiform (GBM), lower grade glioma (LGG), schizophrenia (SCZ), and autism spectrum disorder (ASD) (Supplementary Table 13). We found that many of these sex-specific DEGs lack published findings and that more sex-specific DEGs were identified in brain disorders (ASD and SCZ) than in brain tumors (GBM and LGG) (Fig. 5AB). This finding prompted us to examine whether these sex-specific DEGs are conserved in humans and macaques. We found that most protein-coding DEGs were conserved, but most lncRNA DEGs were not (Fig. 5C), which supports our results described above and suggests that human-specific lncRNAs are associated with sex bias in the brain and sex-biased characteristics of brain diseases. These sex-specific DEGs are enriched for metabolic processes, immune responses, and neurogenesis (Fig. 5D; Supplementary Table 12), which is consistent with the above-described finding that sex-biased genes are enriched in the immune response in humans (Fig. 4B). To reveal where the association occurs in the brain for specific diseases, we compared sex-specific DEGs with sex-biased genes in the DFC and HIP regions. The sex-specific DEGs in the four brain disease samples from DFC and HIP were significantly more enriched with sex-biased genes than non-sex-biased genes in the two regions (i.e., DFC and HIP, Fisher’s exact test, *P* < 0.05; OA > 1.2 for 16/20 sex-specific DEG sets) (Fig. 5E; Supplementary Table 15). An example is *P2RY12*, which is involved in microglial motility and migration [61]. *P2RY12* has sex-biased expression in adolescence and adulthood and is a sex-specific DEG in male GBM. These results suggest that many sex-biased genes influence susceptibility to brain diseases and that more data analyses will help identify sex-specific diagnostic and therapeutic targets of brain diseases.

## Discussion

Sex bias exists in all animals, shows different features across lineages and species, and evolves rapidly across mammalian organs even in closely related species [8]. Data from the UK Biobank database indicate that brain volume and cortical thickness show sex bias [62]; genes show sex-biased expression in the cerebral cortex and in specific developmental periods [5–7]; gene expression shows more cross-species divergence in microglia, astrocytes, and oligodendrocytes than in neurons [59]; and brain diseases have sex bias in incidence, progression, response to treatment, and prognosis [2–4, 63]. These reports may reveal only the tip of the iceberg of sex bias in the brain and call for more comparative analyses of sex-biased gene expression.

A specific issue of sex bias in the brain is the transcriptional regulation of sex-biased genes by lncRNAs. Although the targets of transcription factors have been examined in many studies, the targets of lncRNAs remain unexplored. The transcriptional regulatory function of lncRNAs, the large number of lineage-specific lncRNA genes in mammalian genomes, and the rapid evolution of sex bias in mammals indicate an association between sex bias and lncRNAs. Thus, we postulated that sex bias in the brain is highly human-specific and is strongly affected by human-specific lncRNAs.

To examine this postulation, this study analyzed RNA-seq data from human and macaque brain regions and developmental periods and from patients with brain diseases. Our results indicate that sex-biased gene expression is intensively regulated by species-specific lncRNAs and that sex-biased genes are associated with immune-related functions in the human brain but not in the macaque brain. The former is supported by lncRNA studies  (e.g., lncRNAs are key regulators of genomic imprinting and complex genomic imprinting occurs in the brain) [10, 11, 13, 14, 64]; the latter is supported by findings that gene expression in immune-related microglia, astrocytes, and oligodendrocytes shows more cross-species divergence than in neurons [59]. Our finding that sex-biased genes in human and macaque brains are critically regulated by species-specific lncRNAs reasonably explains the rapid evolution of sex bias [8].

Several notes on the study and results. First, our focus was human/macaque-specific sex-biased gene expression, which is slightly different from sex-biased expression of human/macaque-specific genes. Second, on the basis of our results and the report that approximately one-third of human lncRNAs are primate-specific [65], it is sensible to infer that the ratio of human-specific lncRNA genes is greater in sex-biased genes in the brain than in the whole genome. This high ratio has multiple implications: it indicates that human-specific lncRNAs contribute extraordinarily to sex bias in the brain and suggests that species-specific lncRNAs are a driving force for the rapid evolution of sex bias. Third, since we did not examine other monkeys and apes, genes in humans but not in macaques are not strictly human-specific, and equally, genes in macaques but not in humans are not strictly macaque-specific. Fourth, caution is needed when interpreting the proximity of sex-biased lncRNAs to their targets (Supplementary Table 10) because little is known about whether a lncRNA prefers functioning *in cis* or *in trans*. Additionally, caution is needed when sex-biased genes in the fetal period are interpreted because cells are highly heterogeneous during this period.

Our results also raise new questions. First, multiple studies have reported sex differences in immune responses, for which multiple animal models of diseases have been developed [66, 67]. The rapid evolution of sex bias, the high lineage specificity of lncRNA genes, and the intensive regulation of sex-biased genes by species-specific lncRNAs together call for the cautious use of mouse and rat models of human diseases. Second, the evolutionary selection of new genes and mutations may have promoted human brain evolution but also made humans susceptible to some brain diseases [68, 69]. This selection at the genome level comes with trade-offs [70], and we postulate that sex-biased gene expression is a sort of selection at the transcriptome level and that this selection may also come with certain trade-offs. If so, what sex-biased genes are the primary targets and what sex-biased genes are the consequent trade-off are interesting questions, calling for more analyses that integrate and explore genomic, transcriptomic, phenotypic, and clinical data.

## Conclusions

Human-specific genes greatly cast the sex bias in the brain. Since human-specific sex-biased protein-coding genes are enriched for immune-related functions and human-specific sex-biased lncRNAs regulate sex-biased genes that are enriched for immune-related functions, human-specific genes greatly determine sex bias in the brain and the relationship between sex bias in the brain and brain diseases. The high proportions of lineage-specific lncRNAs in mammalian genomes indicate that sex biases may have evolved rapidly in the brain and other organs.

## Electronic supplementary material

Below is the link to the electronic supplementary material.

Supplementary Material 1

Supplementary Material 2

## Data Availability

No datasets were generated or analysed during the current study.
