# Supplementary material for: Human-specific protein-coding and lncRNA genes cast sex-biased genes in the brain and their relationships with brain diseases
Source: Biol Sex Differ. 2024 Oct 29;15:86. doi: 10.1186/s13293-024-00659-3 (PMC11520681; doi:10.1186/s13293-024-00659-3)
Supplement: Supplementary file 1 — Supplementary Material 1 [file 13293_2024_659_MOESM1_ESM.pdf]

Human-specific protein-coding and lncRNA genes cast sex-biased genes in the brain and the relationships with brain diseases

Sha He[1#], Xuecong Zhang[1#], Hao Zhu[1,2,3*]

1 Bioinformatics Section, School of Basic Medical Sciences, Southern Medical University, Guangzhou, 510515, China

2 Guangdong-Hong Kong-Macao Greater Bay Area Center for Brain Science and Brain-Inspired Intelligence, Southern Medical University, Guangzhou, 510515, China

3 Guangdong Provincial Key Lab of Single Cell Technology and Application, Southern Medical University, Guangzhou, 510515, China

# These authors contributed equally to the work.

* Corresponding author: zhuhao@smu.edu.cn (H.Z.)

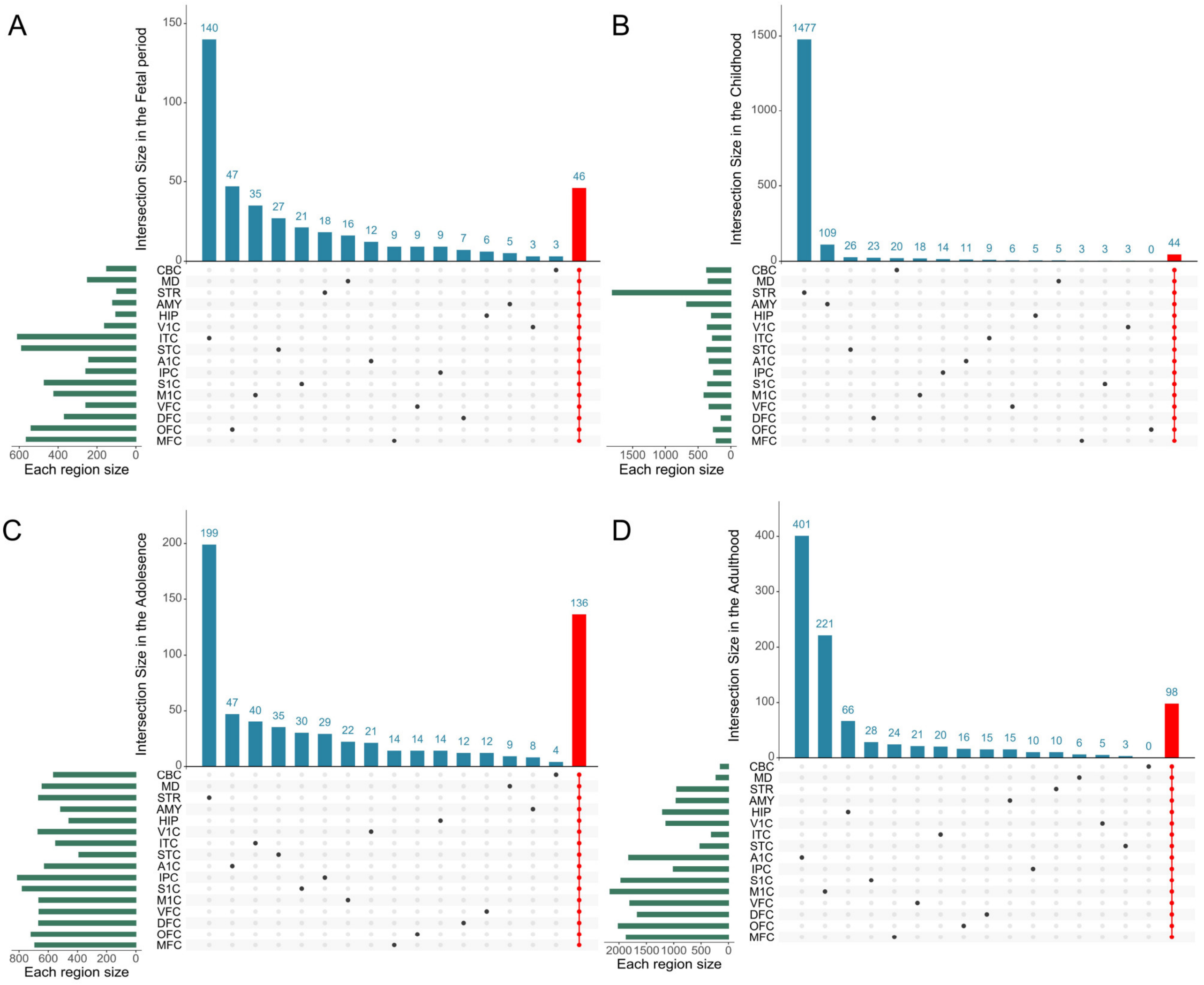

Supplementary Figure 1. The numbers of region-specific sex-biased genes in the human brain. (A) In fetus. (B) In childhood. (C) In adolescence. (D) In adulthood.

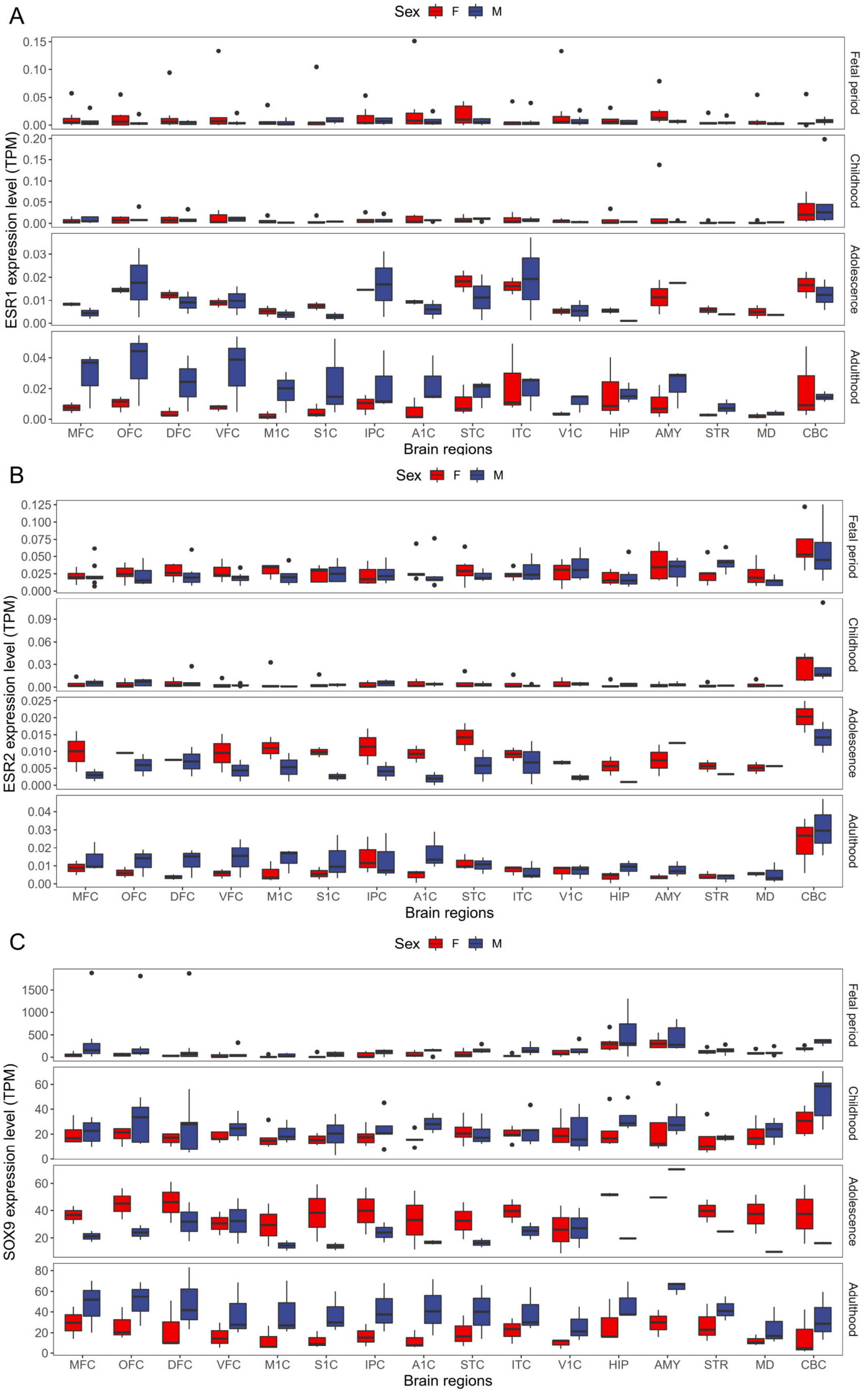

Supplementary Figure 2. The box plots show the expression level of genes encoding ESR1 (A), ESR2 (B), and SOX9 (C) in the 16 human brain regions across four developmental periods.

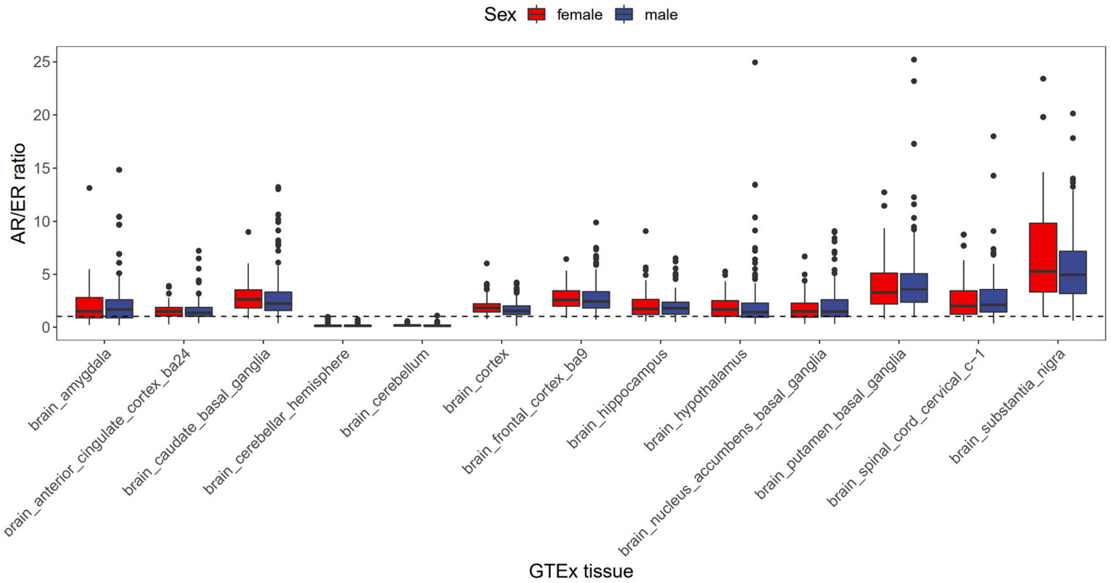

Supplementary Figure 3. The AR/ER expression ratio in 13 human brain regions (GTEx RNA-seq data).

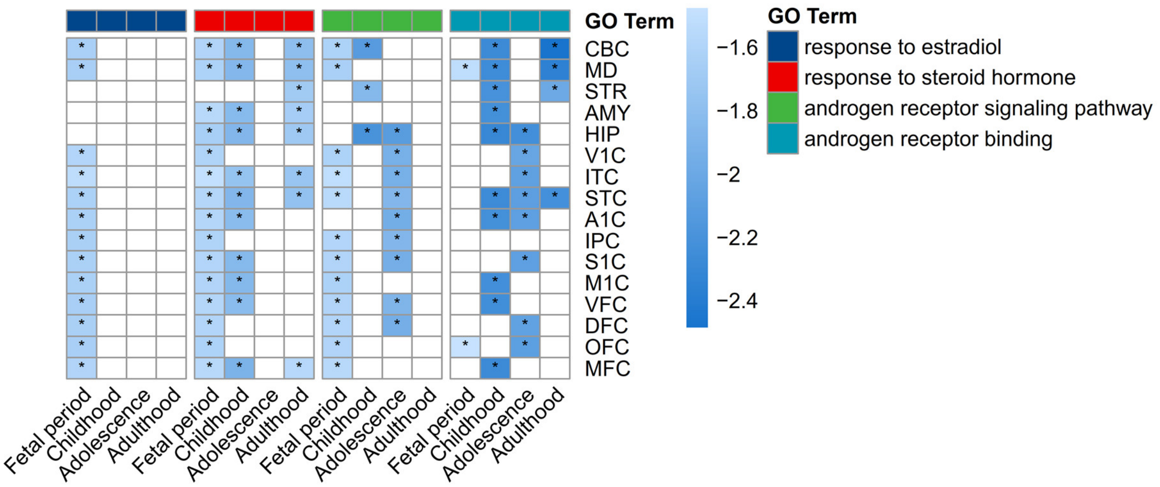

Supplementary Figure 4. The enrichment of sex-biased genes for hormone receptor-related GO terms in different regions and periods. Greyscale indicates normalized enrichment score (NES); negative and positive NES indicates male- and female-specific enrichment; "*" indicates significance (*gseGO* in *ClusterProfiler*, FDR<0.05).

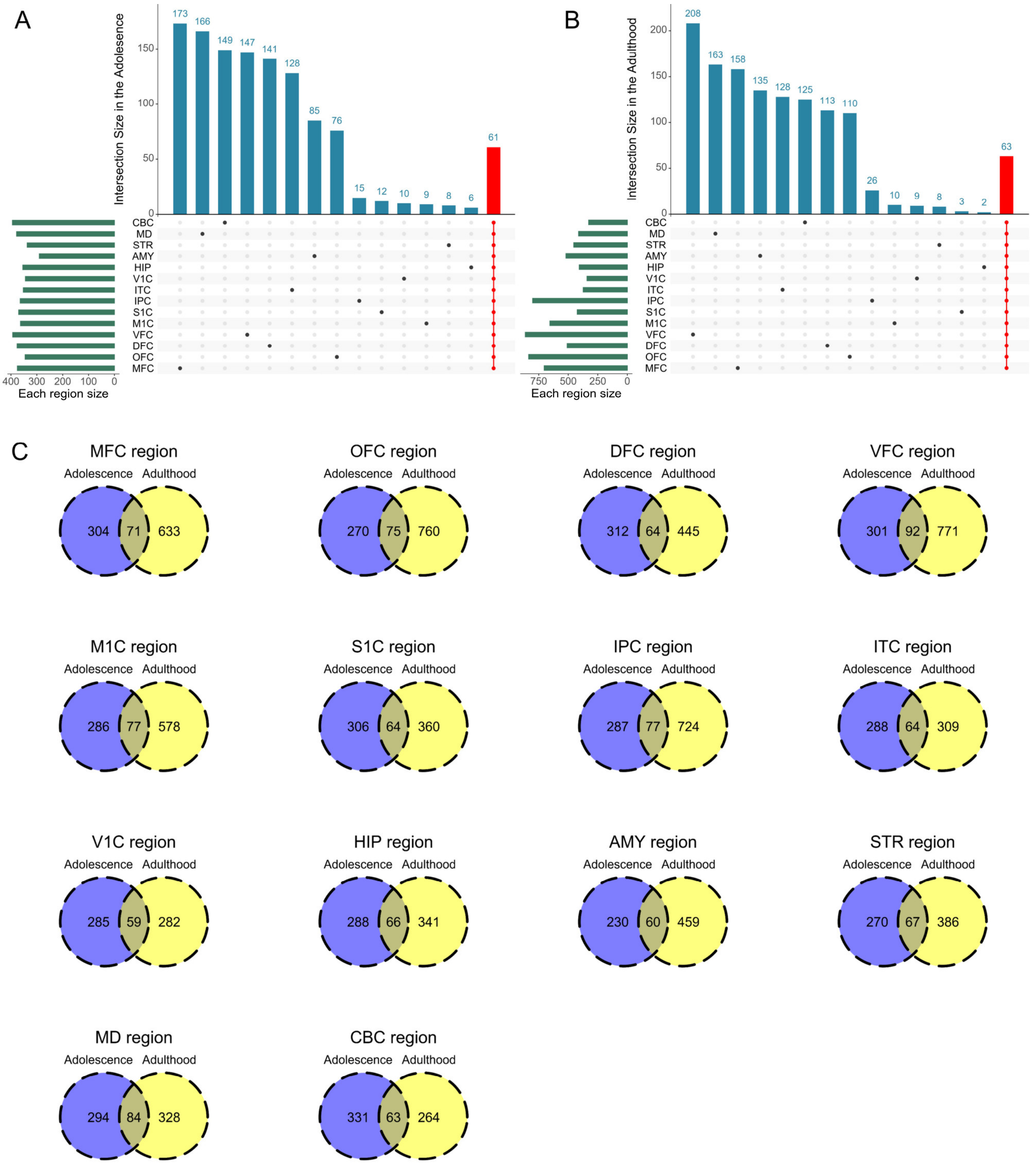

Supplementary Figure 5. Numbers of region-specific sex-biased genes in the macaque brain in two periods. (A) In adolescence. (B) In adulthood. (C) The overlaps of region-specific sex-biased genes between the two periods.

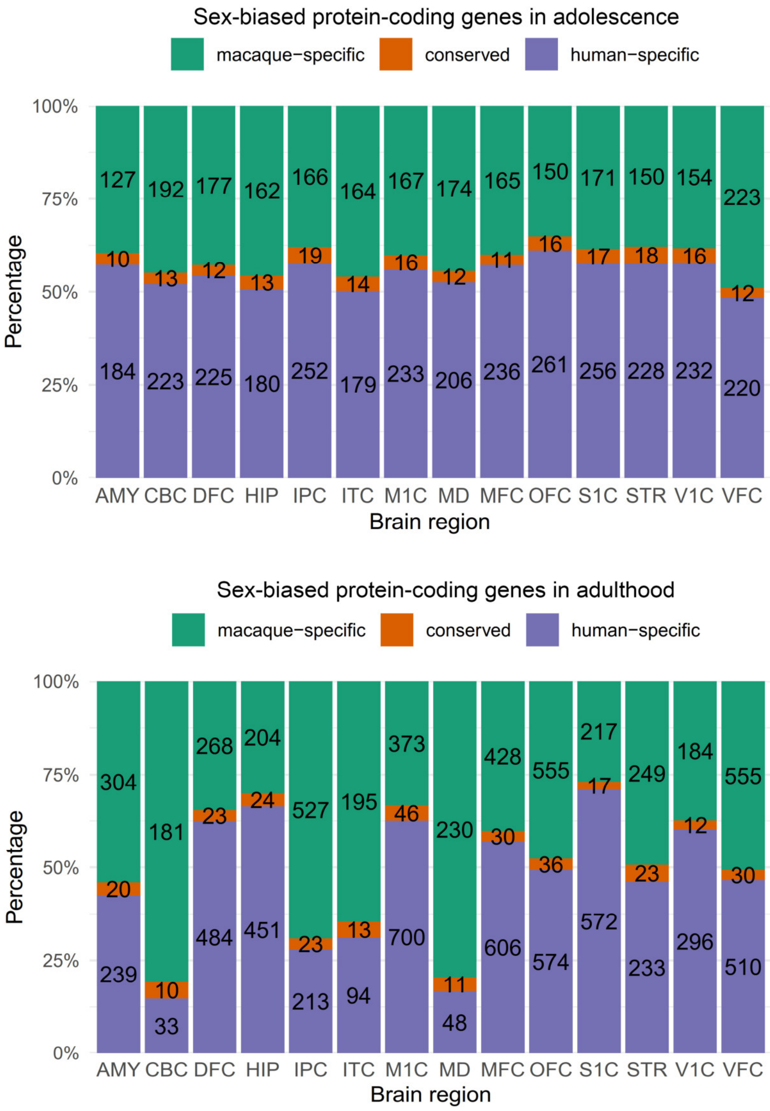

Supplementary Figure 6. Conserved and species-specific sex-biased protein-coding genes in the 14 human and macaque brain regions in adolescence and adulthood.

GO:BP		stats	
Term name	Term ID	p_{adj}	$-\log_{10}(p_{adj})$ (0 – ≤16)
MHC protein complex assembly	GO:0002396	5.745×10^{-3}	
MHC class II protein complex assembly	GO:0002399	5.745×10^{-3}	
peptide antigen assembly with MHC protein complex	GO:0002501	5.745×10^{-3}	
peptide antigen assembly with MHC class II protein complex	GO:0002503	5.745×10^{-3}	
negative regulation of viral life cycle	GO:1903901	1.138×10^{-2}	
antigen processing and presentation of exogenous peptide ...	GO:0019886	1.138×10^{-2}	
antigen processing and presentation of peptide antigen via ...	GO:0002495	1.174×10^{-2}	
antigen processing and presentation of peptide or polysacc...	GO:0002504	1.174×10^{-2}	
establishment of skin barrier	GO:0061436	1.174×10^{-2}	
antigen processing and presentation of exogenous peptide ...	GO:0002478	1.179×10^{-2}	
antigen processing and presentation of exogenous antigen	GO:0019884	1.642×10^{-2}	
monounsaturated fatty acid catabolic process	GO:1903965	2.291×10^{-2}	
antigen processing and presentation of peptide antigen	GO:0048002	2.904×10^{-2}	
positive regulation of immune response	GO:0050778	3.026×10^{-2}	
cell envelope organization	GO:0043163	3.054×10^{-2}	
cytotoxic T cell pyroptotic process	GO:1902483	3.054×10^{-2}	
monounsaturated fatty acid metabolic process	GO:1903964	3.054×10^{-2}	
positive regulation of type III interferon production	GO:0034346	3.054×10^{-2}	
negative regulation of viral process	GO:0048525	3.855×10^{-2}	
B cell affinity maturation	GO:0002344	3.925×10^{-2}	
peripheral B cell selection	GO:0002343	3.925×10^{-2}	
regulation of immune response	GO:0050776	4.104×10^{-2}	
antigen processing and presentation	GO:0019882	4.578×10^{-2}	
positive regulation of RNA polymerase II regulatory region ...	GO:1905636	4.578×10^{-2}	
B cell selection	GO:0002339	4.894×10^{-2}	
medium-chain fatty acid catabolic process	GO:0051793	4.894×10^{-2}	
type III interferon production	GO:0034343	4.894×10^{-2}	
skin epidermis development	GO:0098773	4.894×10^{-2}	
regulation of type III interferon production	GO:0034344	4.894×10^{-2}	

Supplementary Figure 7. Genes in humans but not in macaques are enriched for immune-related GO terms (*gProfiler*, Benjamini-Hochberg FDR<0.05).
